# Medicinal plants used by inhabitants of the Shigar Valley, Baltistan region of Karakorum range-Pakistan

**DOI:** 10.1186/s13002-017-0172-9

**Published:** 2017-09-25

**Authors:** Zaheer Abbas, Shujaul Mulk Khan, Jan Alam, Sher Wali Khan, Arshad Mehmood Abbasi

**Affiliations:** 1 Department of Botany, Hazara University, Mansehra, Pakistan; 2 Department of Plant Sciences, Quaid-i-Azam University, Islamabad, Pakistan; 3 Department of Biological Sciences, Karakorum International University, Gilgit, Baltistan Pakistan; 4 Department of Environmental Sciences, COMSATS, Abbottabad, Pakistan

**Keywords:** Medicinal plants, Shigar Valley, Karakorum, Mountain, Baltistan

## Abstract

**Background:**

The inhabitants of mountainous terrains depend on folk therapies to treat various ailments; however lack of plant based research and geographical constraints set the traditional knowledge in jeopardy. Present study is the first documentation on traditional uses of plant species by the inhabitants of the Shigar Valley, Karakorum Range, Northern Pakistan.

**Method:**

Ethnobotanical data were collected over a period from July, 2013 to October, 2016 from 84 respondents, using semi structured questionnaire. Quantitative indices such as relative frequency citation (RFCs) and fidelity level (FL) were intended to evaluate the importance of medicinal plant species.

**Results:**

In total 84 plant species belonging to 36 families and 72 genera were recorded. Fabaceae was dominant with 7 species, followed by Asteraceae, Lamiaceae and Rosacea (6 species each). Leaves, root, flowers, seeds and fruits were the frequently utilized plant parts, whereas among drug formulations, decoction (49%) was ranked first. Majority of the plant species were used to treat abdominal, respiratory and dermal ailments (31, 12 and 12, respectively). RFCs value ranged 0.477 to 0.11 for *Tanacetum falconeri* and *Allium carolinianum,* respectively; while *Hippophe rhamnoides* and *Thymus linearis* depicted 100% FL. Comparative assessment with previous reports revealed that traditional uses of 26% plant species counting *Hedyserum falconeri, Aconitum violoceum* var. *weileri, Arnebia guttata, Biebersteinia odora, Clematis alpine* var. *sibirica, Corydalis adiantifolia* and *Saussurea simpsoniana* were reported for the first time.

**Conclusion:**

The endemic medicinal plant species and traditional knowledge of Balti community living in extremely high mountains area were explored for the first time. A comprehensive survey of this region could be significant to drive the existing knowledge in market circuit with sustainable collection, and to evaluate economic potential of the plant species. Additionally, social livelihood could be reinforced through establishing collection sites, transformation and drying centres for micro and macro marketing of medicinal plant species.

**Graphical abstract:**

Plants and people interaction in the Karakorum Mountains
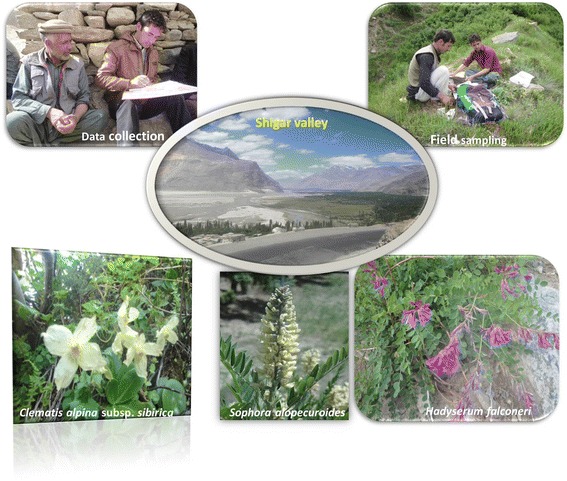

## Background

Mountain landforms cover about one quarter of the land surface and host 12% world’s population [37]. These landforms have great influence on climatic, biological, ethnic, cultural and linguistic diversity of any region. In Pakistan, substantial rural population is living in the mountain ranges of Karakorum, Himalaya and Hindu Kush. The Karakorum ranges frame deep incised valleys in the extreme north of Pakistan, and provide several services to dwellers such as timber, fuel wood, fodder, herbal medicines etc. Because, harsh climate, remoteness and difficult access hamper development in basic services particularly, education and health [55]. Therefore, mountain people are considered as the most poorest and deprived population. Furthermore, the inhabitants of high mountain areas are more susceptible to various diseases owing to unsympathetic mountains’ environment with unexpected fluctuations in seasonal temperature, light intensity, ultraviolet (UV) radiation and poor domestic hygiene [3]. The health facilities provided by government and non-governmental organizations (NGOs) are next to nothing for the inhabitants living in these remote areas. Consequently, in such circumstances plant based traditional therapies are the primary health care source to mitigate various health disorders.

Baltistan is an archetypal mountainous region of the Northern Pakistan with average altitude of 3555 m above sea level. Historically, it has often been referred as “Western Tibet” or “Little Tibet” [6, 58]. The territories of the Baltistan region lie sparsely at acclivities and in deep mountains of Karakorum and Himalaya with unique landscape, climate, flora and fauna. However, remoteness, difficult access and inadequate funding may be the major handicaps to conduct field survey in these areas. Only few workers [11, 12, 21], have conducted ethnobotanical survey in some parts of Northern Pakistan. Therefore, very limited ethno-botanical literature is available in the region [20, 31]. Shigar valley is located in the Karakorum Ranges, and is the home of various peaks (including K2), glaciers and hot springs, which have always been the most preferred tracking places for visitors across the country and abroad. Ethno-botany is a recently introduced and rapidly flourishing field in this region, and is gaining adequate attention by researchers. Although, various ethnobotanical surveys have be conducted in different parts of Pakistan. However, Northern parts of country are still poorly explored. Therefore, present survey aimed to provide, the first inventory on ethno-pharmacological application of medicinal plant species used by the inhabitants of Balti community of Shigar valley, Karakorum Mountains-Pakistan.

## Methods

### Study area

**Fig. 1:**
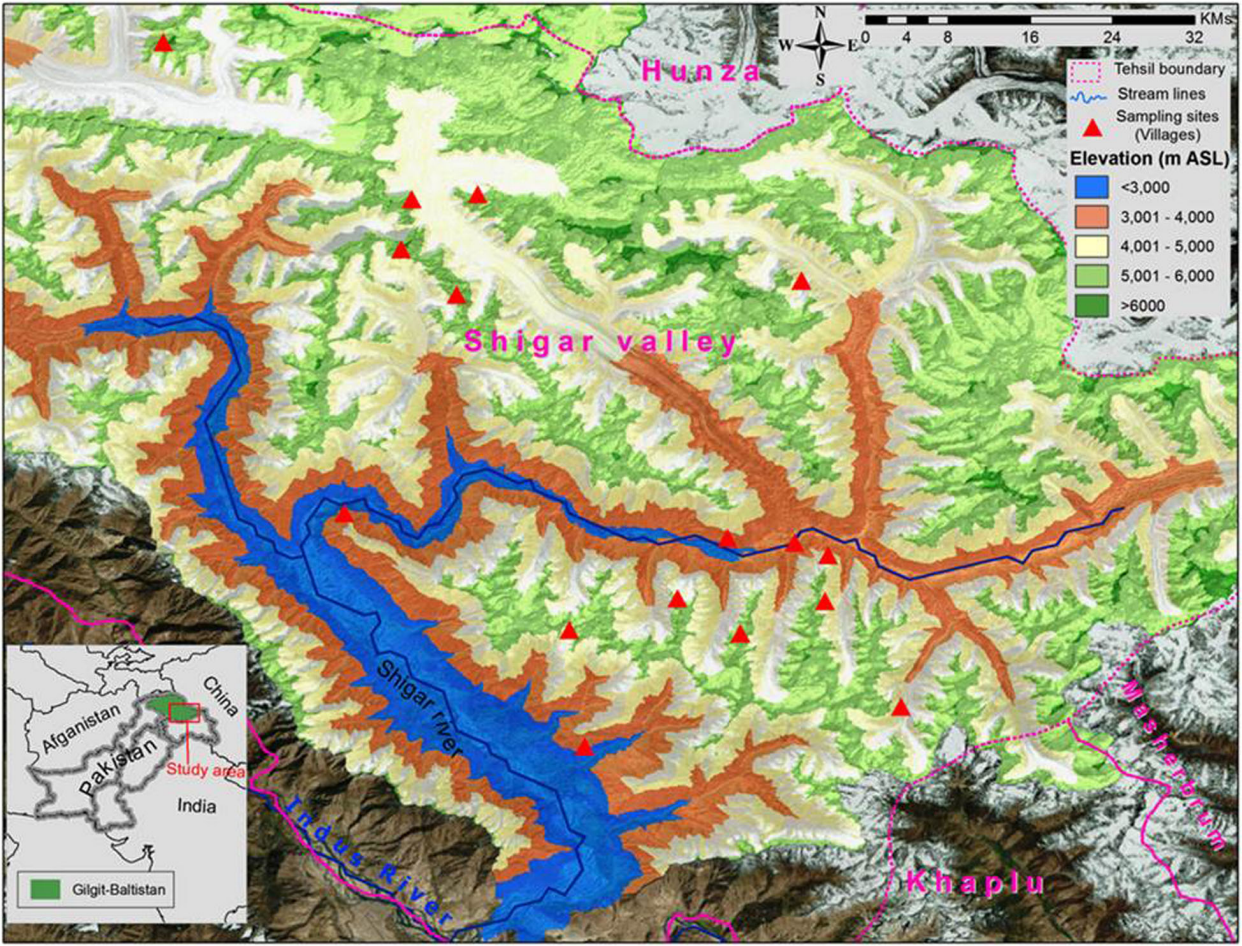
Map of the study area showing survey sites

Shigar Valley is a part of the central Karakorum ranges situated in the north of Skardu town at right bank of the river Indus (Fig. 1). It lies at 25° 25′32″ N latitude and 75° 42′59″E longitude and covers an area of 4373 sq. km with altitudinal amplitudes of 2, 260 to 8611 m above sea level [45]. It borders with China fenced by K2 (Godwin Austin) between the territories [47]. The highest zone above 6000 m encompasses maximum ridges and peaks including K2 (8611 m), Broad Peak (8047 m), Angel Peak (6858 m) and Skil Brum (7360 m). Settlements are distributed in small villages on alluvial fans, terraces and gentle slopes at altitude of 2300 m (Marapi), 2790 m (Arando) and 3050 m (Askole). The valley experiences dry, hot and sunny summer with intensive radiation providing very short growing season for native flora [48].

Shigar Valley was ruled by Raja dynasty ‘Amacha’ and the period of Raja ruling system was known as Chou-Tus (Raja’s period) in Balti dialect [6]. This autocratic system remained in power till the middle of nineteenth century (H. [26, 46]). The presence of human community in Baltistan is prior to the birth of Guatama Budha (563 BC). The indigenous people of the valley have migrated from different regions i.e. Ladakh (Indian Kashmir), Tibet (China) and Hunza-Nagar via mountainous tracks [60]. These migrants have intermixed culture and arose as single Balti ethnic group due to dominant Ladakhi and Tibetan Balti speakers. They speak an archaic non-written Tibetan dialect called Balti [14]. Balti caste has a number of lineage sub-groups (Clan), which are also known as mi-schir (pronounce as mee-ser) in local dialects. They are very traditional and still tightly knotted with Balti culture in constructions, house scheming, livestock homes; dressing, agricultural activities, domestic and farming tools, games and rustic practices.

### Data collection

**Fig. 2:**
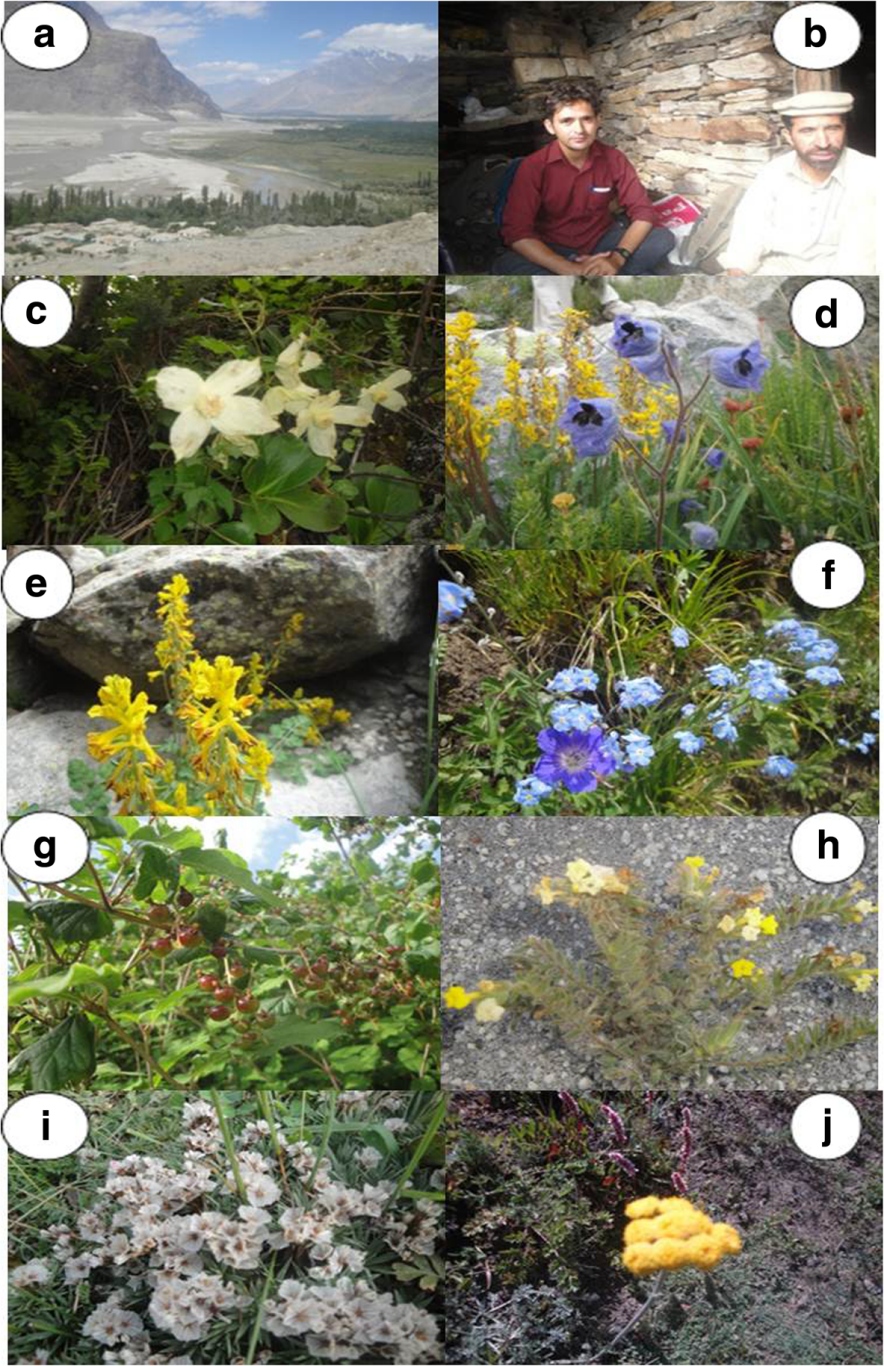
**a** Landscape of the Shigar valley **b** Principal author with local guide in shepherd’s house in alpine pasture **c** *Clematis alpina* var. s*ibirica* a very rare medicinal plant of the study area **d** *Delphinium brunonianum* a high altitude medicinal species **e** *Corydalis adiantifolia* a newly reported medicinal plant from the region **f** *Myosotis alpestris* commonly distributed species from sub alpine to alpine zone **g** *Ribes himalayense* a medicinal shrub and local wild fruit **h** *Arnebia guttataa* lower altitude rare species **i** *Acantholimon lycopodioides* **j** *Tanacetum falconeri* common high altitude species

Ethnobotanical survey was conducted from July, 2013 to October, 2016 in seventeen sites of the study area (Fig. 2). Field trips and interviews were planned in early spring i.e. March to June (off vegetation season), while keeping in mind the cold climate and short vegetation season of the area. Eighty four people were interviewed in Balti dialect without distinction of gender after seeking the consent, while semi structured questionnaire as explained previously [16, 41] was used to collect data. Interviews were taken in houses, people gathering place, mosques and Jamias (second time Islamic schools). After inquiring the demographic background of the respondents, information were collected on local name of plant species, part used, drug preparation, mode of administration and ailments treated. Afterwards a separate list of reported medicinal plant species with local name was developed by sorting questionnaires along with their ethno-medicinal uses.

### Sampling, preservation and identification

The plant sampling was done in summer. Each specimen was tagged with its local name in the field (Fig. 2), prostrated in blotting papers and kept in filed plant presser. The perplexing plant species were confirmed by local respondents showing plant materials and/or their pictures. Dried specimens were poisoned using Mercuric chloride and absolute alcohol (2 g mercuric chloride +1000 mL absolute alcohol), then mounted herbarium sheets [39]. Preserved specimens were identified by Taxonomists of Hazara University Herbarium, Mansehra Khyber-Pukhtunkhwa Pakistan, Karakorum International University Gilgit-Baltistan, Pakistan and with the help of available literature i.e. Flora of Pakistan [8, 43] and the flora of China. The botanical names and plant families were confirmed by angiosperm phylogeny group and The Plant List. The plant specimens were given the voucher numbers and kept in the Herbarium of Hazara University, Mansehra, Pakistan.

### Ethno-botanical data analysis

Data were analysed using ethnobotanical indices i.e. relative frequency of citation (RFCs) and fidelity level (FL) in order to evaluate the importance of the recorded species.

#### Relative frequency citation (RFCs)

Reveals the importance of each species and is calculated on the basis of the frequency of citation ‘FC’ (the number of informants mentioning the use of species), using formula as described before [51]. The FC value is divided by total number of informants participating in the survey (N), without considering the use-categories

$$ \mathrm{RFCs}=\frac{FCs}{N} $$

Where, FCs is the number of informants who mentioned the use of a plant species and N is the total number of informants.

#### Fidelity level (FL)

Is the ratio between number of informants who mentioned the use of a plant species for a particular purpose and total number of informants who mentioned the use of that plant species for any purpose (regardless the category). FL indicates the percentage of informants claiming the use of plant species for same major purpose. The Fl of the species was calculated using the method as adopted by [7]. High FL values (100%), are obtained for plant species, where almost all uses refer to same purpose. The low FLs are usually obtained for plants that are used for numerous purposes.

$$ \mathrm{FL}\ \left(\%\right)=\frac{lp}{lu}\times 100 $$

*Ip* is the number of informants who independently suggested the use of a plant species for a particular disease and *Iu* is the total number of informants who mentioned the same plant for any disease.

## Results and discussions

### Demographic feature and indigenous knowledge

In total 84 respondents including 73.80% male and 19.67% female were interviewed to collect data on medicinal uses of plant species from seventeen villages without gender distinction (Table 1). Due to Islamic instructions, communal restrictions and isolated society, usually female avoid to participate and hare knowledge, because of Islamic instructions, communal limits and isolated society. The informants were categorized in three age groups i.e. 20–40 year, 40–60 year and above the age 60 years. The middle age people (40–60 years old) had more indigenous knowledge compared to other age groups. This may be due to lack of interest in early age people about traditional remedies, and loss of mammary in elderly people because of age factor.

**Table 1:** Demographic feature of respondents in the Shigar Valley

Variables	Categories	Number of persons	Percentage
Sex Ratio	Women	22	25.19
	Men	62	73.80
Age Groups	Between 20 and 40 years	21	29.50
	Between 40 and 60 years	47	45.90
	Above 60 years	16	24.59
Education Level	Illiterate	56	75.40
	Primary	9	4.920
	Middle	5	6.550
	High School	7	4.920
	Graduate	6	3.280
	Masters	1	4.920
Social Livelihoods	Farmers	56	62.29
	Shepherds	7	8.190
	Wood cutters	4	6.550
	Gems (mining)	9	13.11
	Healers	4	4.920
	Job Holders	4	4.920
Life type	Town area	28	42.62
	Elevated areas	56	57.37

As far as education of the informants was concerned, majority of the respondents (75.40%) were uneducated, however 24.60% were literate: having primary to masters level of education. Local inhabitants of the valley have different sources for survival. Most of them are farmers, shepherds, wood cutters, gemstone workers and job holders. It was interesting to know that local healers treat villagers and extend their traditional therapeutic knowledge among other people free of cost. The people of less developed and elevated zone possess adequate knowledge on medicinal use of native flora compared to those who live in towns. However, rapid modernization and effortless access to allopathic medicines might be the main causes that are diminishing the traditional knowledge of the dwellers.

### Ethno-floral diversity, availability and habit

A total of 84 medicinal plant species belonging to 72 genera and 36 families were used by the inhabitants of the area to treat various health disorders. An enumeration of all recorded species including botanical name, local name, voucher specimen number, family, habit, availability, locality, parts used and drug description is provided in (Table 2). Fabaceae was the leading family with 7 species, followed by Asteraceae, Lamiaceae and Rosacea each was represented by 6 species. The therapeutic significant of the first four families may be associated with common distribution of species belong to these families in the study area. Asteraceae is one of the largest families in the flora of Pakistan, and its prevalent distribution throughout the country may be the reason behind being dominant. Likewise, same family been reported as a leading family in the previous studies conducted in surrounding areas of Shigar Valley [1, 12, 21]. However, Khan (2007) reported Rosaceae as the most prevailing family from various valleys of Himalaya and Karakorum ranges of mountains [29, 31]. These findings indicate the ample indigenous knowledge, varied selection and rich diversity of medicinal flora of the region.

**Table 2:** Family wise distribution of medicinal plants in study area

Family	Number of Species	Percentage%	Family	Number of Species	Percentage%
Alliaceae	2	2.380	Juglandaceae	1	1.190
Apiaceae	5	5.950	Lamiaceae	6	7.140
Asteraceae	6	7.140	Moraceae	1	1.190
Berberidaceae	3	3.570	Oleaceae	1	1.190
Betulaceae	1	1.190	Papaveraceae	1	1.190
Biebersteiniaceae	1	1.190	Parnassiaceae	1	1.190
Boraginaceae	3	3.570	Plantaginaceae	1	1.190
Brassicaceae	2	2.380	Plumbaginaceae	1	1.190
Capparidaceae	1	1.190	Poaceae	3	3.570
Chenopodiaceae	1	1.190	Polygonaceae	5	5.950
Cupressaceae	1	1.190	Punicaceae	1	1.190
Cuscutaceae	1	1.190	Ranunculaceae	6	7.140
Elaeagnaceae	2	2.380	Rosaceae	6	7.140
Ephedraceae	1	1.190	Salicaceae	2	2.380
Equisetaceae	1	1.190	Saxifragaceae	1	1.190
Fabaceae	7	8.330	Solanaceae	2	2.380
Fumariaceae	1	1.190	Urticaceae	1	1.190
Gentianaceae	1	1.190	Zygophyllaceae	1	1.190
Glossulariaceae	3	3.570			

**Fig. 3:**
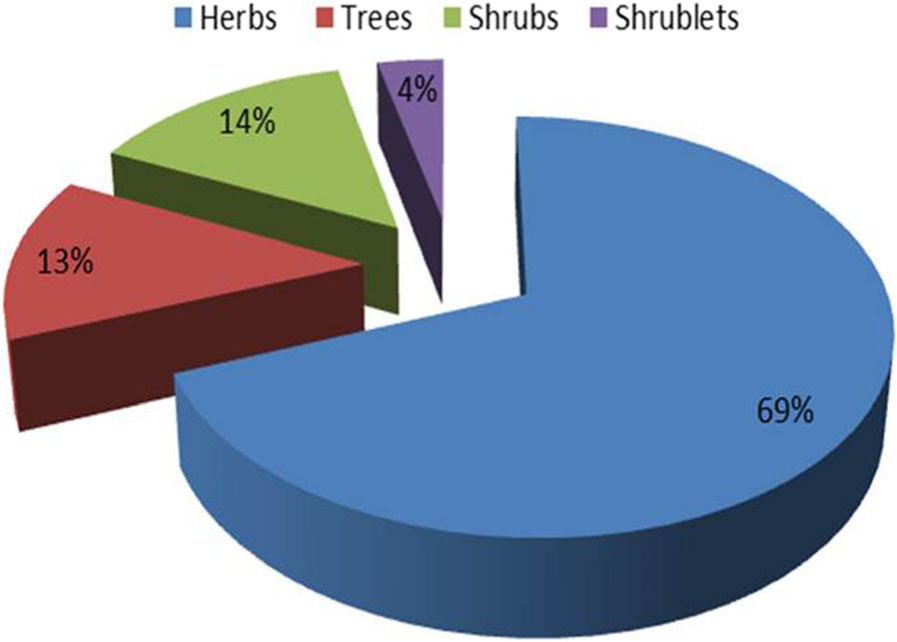
Growth forms of used medicinal species

Inhabitants of the Shigar Valley use cultivated and wild plant species (73.80 and 22.19%, respectively) in traditional drug therapies, which is in agreement with previous study conducted in Haramosh and Bugrote valleys, in Gilgit-Pakistan [34]. Except for *Equisetum arvense* and *Ephedra gerardiana* rest of the species were angiosperms. Herbs were the dominant with 69% contribution (Fig. 3), followed by shrubs, trees and shrub lets (14, 13 and 4%, respectively). The climatic conditions, wide distribution and easy access may be the reasons behind prevailed herbaceous habit in the area [2, 3, 35].

### Part(s) used, drug preparation and administration

**Fig. 4:**
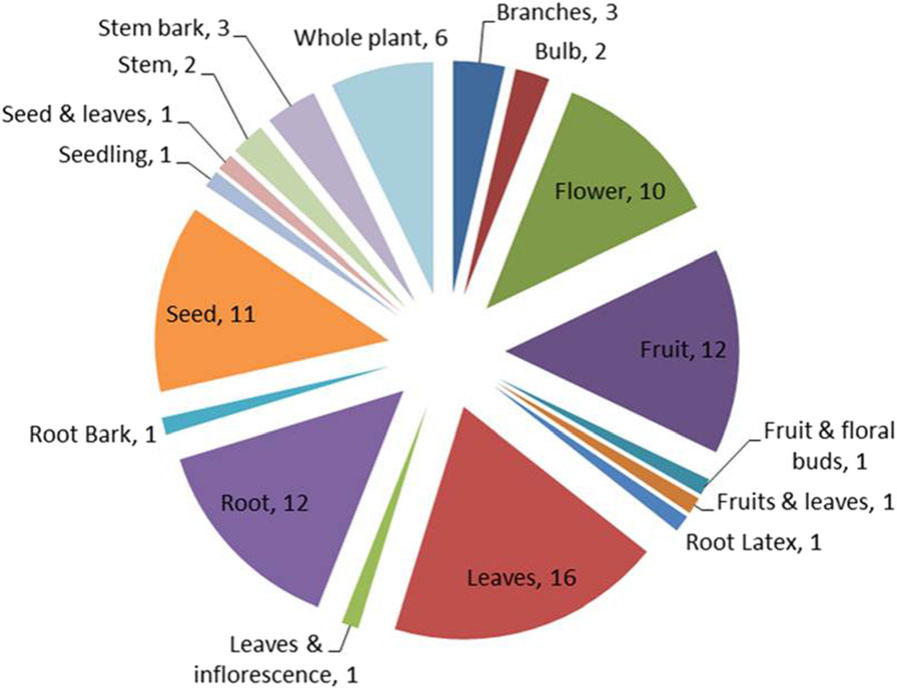
Plant parts used in various disease treatments

The plant part(s) and their use number i.e. use more than one part of plant as drug source, depend upon the availability and indigenous knowledge of local community. The parts of plant species used were grouped in seventeen (17) categories on the basis of their types and number such as: as branches, bulb, flower, fruit, leaves, root, seed, seedling and whole plant were one part used categories (Fig. 4), whereas fruit & floral buds (*Prunus armeniaca*), fruits & leaves (*Hippophe rhamnoides subsp. turkestanica*), inflorescence, root latex and powder (*Ferula jaeschkeana*), leaves & inflorescence *(Salix alba*), root bark & fruit (*Rosa webbiana*), seed & leaves (*Pimpinella diversifolia*) and, stem bark and seed (*Fraxinus xanthoxyloides*) were two parts used categories. Leaves were the most frequently used plant parts (16 species), followed by fruits (12), root (12) seed (11) and flowers (10).

**Fig. 5:**
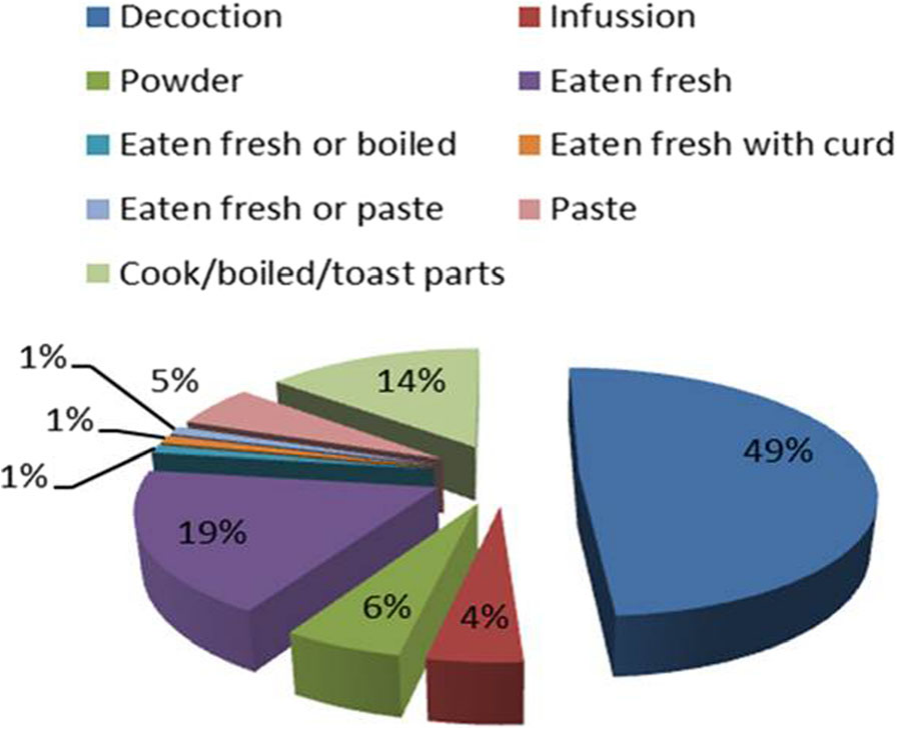
Different modes of drug formulation

**Fig. 6:**
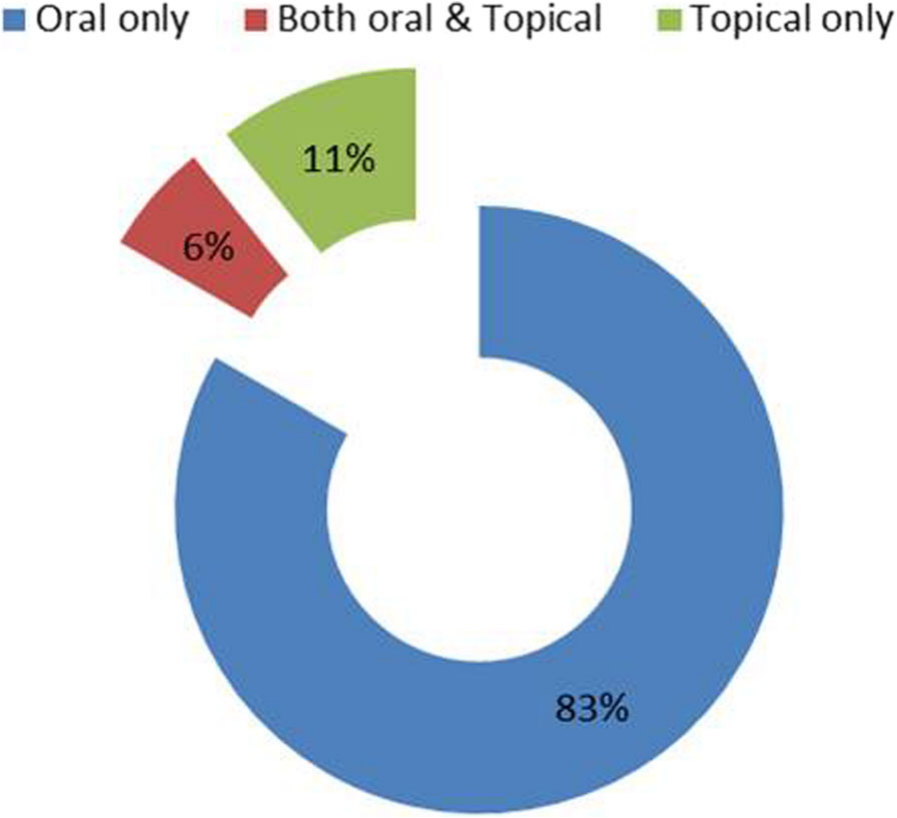
Mode of administration

The inhabitants of the study area use nine types of drug formulation as mentioned in Fig. 5, to treat various ailments. Among these, decoction was dominant (45 medications), followed plant part(s) eaten fresh (17 medications), cook/boiled/toasted (13 medications), powder and paste (5 medications each) and infusion (4medications). The modes of drug administration were divided in to three groups (Fig. 6). Around 83% recipes were taken orally, 11% were applied topically and 6% were used as oral and topical.

### Classification of diseases and traditional therapies

**Fig. 7:**
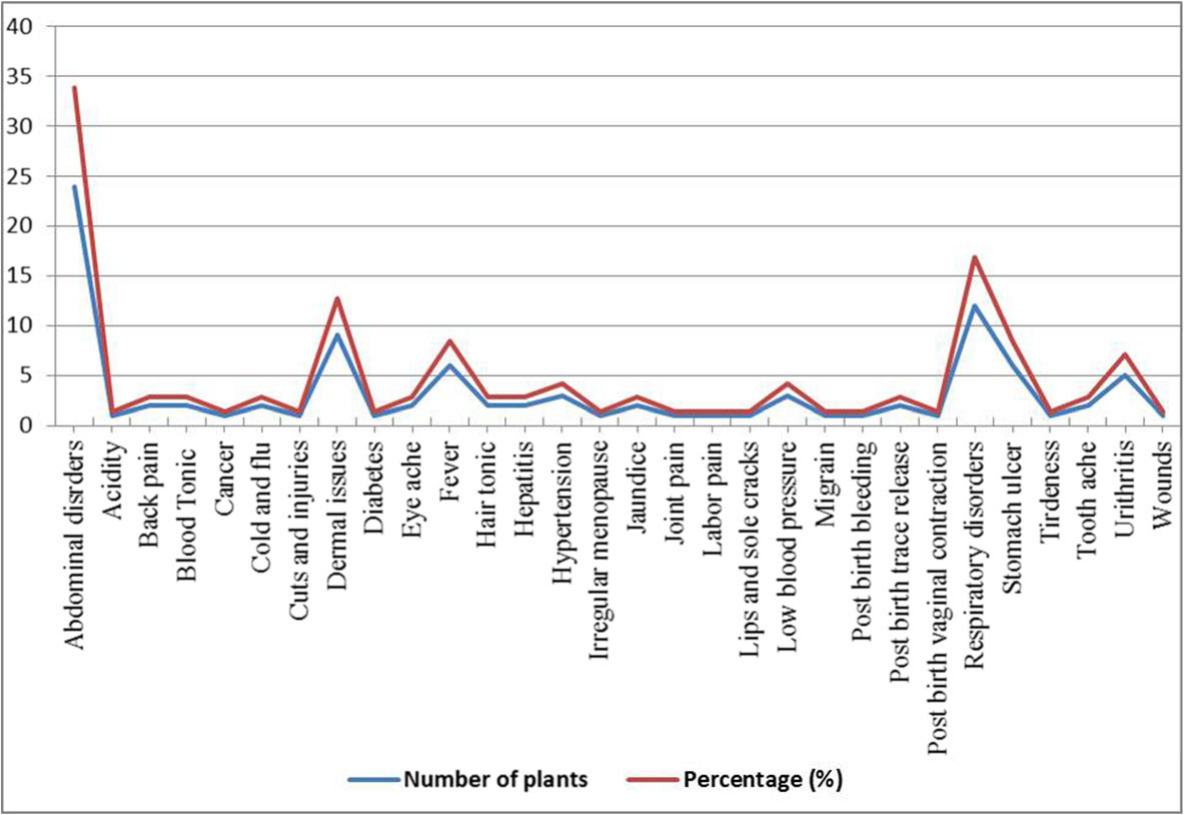
Ailments categories and number of plant used

**Fig. 8:**
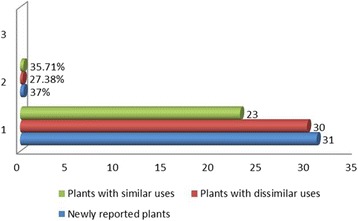
Report statuses of the documented plants

The life of rural peoples, particularly those who live in high mountain areas is very tough. The continuous effort of these people in order to survive in the social hierarchies makes them vulnerable to various types of ailments. The inhabitants of the Shigar Valley use medicinal plant species to treat 30 different ailments (Fig. 7). These ailments were grouped into fourteen categories by *emec* classification method viz. abdominal disorders treated by 31 species, dermal problems (12 species), respiratory disorders (12 species), miscellaneous including cold, cough, fever, migraine, vomiting (12 species), menstrual and pregnancy problem (6 species), cardiovascular disorders (6 species), tonics (5 species), urinary tract disorders (5 species), hepatic disorders (4 species), bones and joints issues (3 species), optical disorders (2 species), tooth problems (2 species), cancer (1 species) and diabetes (1 species). These observations were correlated with previous reports [18, 30, 56]. Gastrointestinal (GIT) disorders (i.e. constipation, indigestion, gastric trouble, dysentery, acidity, and stomach ulcer), skin diseases (pimples, pustules, and ringworm), respiratory tract infections (bronchitis, asthma, pneumonia) and bones/joint ailments (back ache, arthritis) were the common health problems. The gastrointestinal (GI) disorders may attributed to domestic hygienic conditions and dietary routine. Additionally, frequent use of teas, red pepper and less fibrous food could be a reason of GI infections. Likewise, intensive ultraviolet radiations and poorly managed public sanitation may be accountable for the prevalence of dermal problems. Moreover, prolonged harsh and hostile weather and allergens make dwellers vulnerable to get respiratory infections. The bones and joints ailments could be associated with the difficult topography and laborious life style (Fig. 8). Our findings provide an enthusiastic understanding on prevalence and distribution of the public sicknesses. In this context, present work offers imperative idea to frame long term health policies in order to convey health risk, precautions and effective treatment by integrated disease management.

Folk therapies used by the inhabitants of Balti communities were compared with previous work done in Gilgit-Baltistan, other parts of the country and Himalayan communities of India, Nepal and China [40]. The inhabitants of Shigar Valley use *Betula utilis* to treat ringworm, however same species is used against leprosy and earache in Chapursan valley, Hunza [57]. *Delphinium brunonianum* is effective for asthma, gastric trouble and trace release after delivery, but Hussain et al. [21] reported that in central Karakoram National Park this species is used to treat baldness, stomach ache and diarrhoea. *Solanum nigrum* has therapeutic importance of being liver tonic, to alleviate indigestion and eye pain, and to treat skin infections in India and the lesser Himalayas of Pakistan [4], but the inhabitants of Shigar Valley use this species only to treat tooth ache.

The inhabitants Swat Valley use *Artemisia scopria* to treat abdominal worms [19]. Same species has been reported as purgative in Gujrat Pakistan [22] and an effective remedy against hyper-acidic stomach in Zhejiang province, China [15]. However, in Shigar Valley the infusion of *A. scopria* are used to treat diabetes. In Shigar Valley, bulb of *Allium carolinianum* is used to treat gastrointestinal disorders and joint problem, but in Khunjerab National Park, Hunza, this species is used in flu and fever treatment [25]. *Thymus linearis* is used to alleviate abdominal pain and vomiting, while in Astore same species is used to kill abdominal worms [49].

Fruits and leaves of *Hippophe rhamnoides* subs. *Turkestanica* are used in the treatment of gastrointestinal disorders and skin diseases. Same species has been reported to treat cardiac diseases, cancer and stomach ache in Haramosh and Gilgit valleys-Gilgit [33]. Likewise, in Ladakh district of India, this plant is used to treat gynaecological disorders such as irregular menstrual cycles, amenorrhea or dysmenorrhoea [10], and to improve digestion [9]. *Pimpinella diversifolia* is among the most common medicinal herbs in the study area, which is used for abdominal disorders, fever and blood purification. In Lesser Himalayan region of Pakistan and Lao PDR, this species is used to alleviate gas problems and indigestion [5, 17]*. Thalictrum foliosum* is used to cure diarrhoea and loss of appetite in Nepal [24], whereas the inhabitants of Shigar Valley use *T. foliosum* to treat eye ache. Present uses of *Ephedra gerardiana* and *Foeniculum valgare* to treat respiratory and gastro-intestinal disorders, respectively were comparable to previous reports from Rasuwa District, Central Nepal [54] and China [59]. *Rumex nepalensis* is used against stomach pain and itching in Garhwal Himalaya [53], but the inhabitants of the study area use this plant species to treat delivery pain.

The comparative assessment of present applications of medicinal plant species with reported literature revealed strong heterogeneity in folk uses. These findings evidently showed that most of the species are confined in the mountains of Karakorum, which have rarely been reported before. In the regional contrast, our study showed substantial harmony with the work conducted by Hussain et al. in the central Karakorum Range (I. [21]) and to some extent with the ethnobotanical survey carried out in Deosai plateau (Western Himalaya) Baltistan region [11, 12]. Although, both studies were focused on Balti communities, however present evaluation gives strong clues of the variations in ethnobotanical uses with respect to geographical location and difference in vegetation type [27, 28]. 

Our findings also depicted some resemblance with studies conducted in the Karakorum range of Haramosh and Bugrote valleys by ([34]; and [32]). However, no understandable resemblance was observed with the ethnobotanical surveys conducted in other regional communities (Brushiski, Shina and Wakhi) except the work [25] in Khunjerab Hunza, where less than fifteen species were similar to present report. This may be because of floristic resemblance of these two mountainous areas; however different communities hold diverse ethno-flora and related traditional knowledge. Furthermore, change in indigenous knowledge might be linked with difference in area, language and the culture of local communities. Therefore, present assessment pointed out that, phytotherapies of Balti community are diverse and unique in these mountain terrains.

The findings of present study were also compared with previous studies conducted in the Himalayas of India, China and Nepal, which revealed that only few plant species were comparable which include: *Allium carolinianum, Allium cepa, Artemisia scoparia, Berginia stracheyi, Hippophe rhamnoides* and *Thymus linearis* to India, China and Nepal*.* These results may be linked with the floristic and cultural similarity, because analogous to Baltistan; Ladakh is also the home of Balti and Brokpa communities [13, 61], which have similar traditional knowledge on surrounding plant biodiversity. Additionally, due to similar climatic, topographic and edaphic conditions; the flora of study area shares a number of species with Ladakh, Jammu and Kashmir state of India [36].

### Quantitative assessment of ethnobotanical data

Homogeneity in the traditional knowledge of medicinal plants used by the Balti community was evaluated using quantitative indices such as frequency of citation (RFCs) and fidelity level (FL). Relative frequency of citation (RFCs) of the reported species was ranged between 0.049 to 0.377. *Tanacetum falconeri* and *Thymus linearis* exhibited high RFSc value (0.377), followed by *Taraxacum officinale* (0.344) and *Mentha arvensis* (0.327), whereas lowest RFCs value (0.049) was calculated for *Ranunculus repens*. These findings were in agreement to Mutheeswaran et al. [42] in case of *Allium cepa,* whereas disagree in the case of *Tribulus terristris.*

Fidelity level (FL) indicates the most preferred species mentioned by local people to treat a particular disease. According to Lozada et al. information about FL of a species is of significant value compared to other plant based information [38]. The fidelity level of reported species ranged from 28.50 to 100%. Two species i.e. *Hippophe rhamnoides* and *Thymus linearis* depicted 100% fidelity level, whereas lowest FL was calculated for *Foeniculum valgare* (28.50%). These findings were in agreement to [23]. Additionally, *Ribes himalaynse*, *Rosa webbiana*, *Tanacetum falconeri*, *Beibersteinia odora* and *Betula utilis* were the most preferred species with FL more than 90%. The high FL of these species may be attributed to availability, distribution and detail information regarding therapeutic uses, dosage and recipes of these species.

### Novelty and future impact

Present survey is the first comprehensive report on ethnomedicinal uses of plant species in Shigar valley, and reveals that the inhabitants of study area possess ample traditional knowledge on local flora. A careful probe on documented plant species for their medicinal and traditional uses from different areas of Pakistan revealed that, 22 plant species were reported for the first time from the study area, including five species: *Aconitum heterophyllum*, *Salix alba*, *Prunus avium, Ranunculus repens, Populus nigra* and *Malus pumila,* which were new to regional flora. Two endemic species i.e. *Aconitum violoceum* subs. *Weileri* and *Arnebia guttata* were used to treat women sterility, abdominal worm, tonsillitis, inflammation and indigestion by the inhabitants of the study area (Table 3). Among others: *Artemisia santolinifolia* was used against abdominal worms*, Biebersteinia odora* to treat migraine and fever*, Corydalis adiantifolia* as hair tonic, *Clematis alpina* var. *sibirica* and *Desmodium gingeticum* against cough, cold and asthma, *Acantholimon lycopoidioides* for stomach ulcer*, Dracocephalum nutans* to treat asthma, gastric trouble, and post birth trace release, *Ferula jaeskeana* for arthritis, asthma, menopause, *Fraxinus xanthoxyloides* to expel ringworm and tooth ache, *Hedyserum falconeri* in case of indigestion, loss of appetite and constipation, *Myosotis alpestre* to treat asthma, bronchitis, and fever, *Parnassia nubicola* for low blood pressure, gastric trouble, *Saussurea simpsoniana* to treat back ache, fever and dermatitis, *Sophora alopecuroides* against arthritis and *Rumex patientia* for boils and pustules.

**Table 3:** Enumeration of medicinal plant species of Shigar Valley, Baltistan Karakorum, Pakistan

Botanical Name/Local name /Voucher number	Family	Availability	Habit	Part (s) Used	Ailment (s) cured	Application	Formulation	RFCs	FL (%)
*Allium cepa* L. /chong/SP-124	Alliaceae	Cult.	Herb	Bulb	Indigestion and Vomiting	Oral	Cook in ash	0.119	60.00
*Allium carolinianum* DC /Broq chong/SP-74	Alliaceae	Wild	Herb	Bulb	Abdominal pain	Oral	Cook in ash	0.119	80.00
*Foeniculum valgare* Mill. /Badian/Sp-48	Apiaceae	Cult.	Herb	Seed	Constipation and gastric trouble	Oral	Decoction	0.167	28.57
*Pimpinella diversifolia* DC. /Kohniod/SP-135	Apiaceae	Wild	Herb	Seed/Leaves	Fever, abdominal pain and as blood tonic	Oral	Decoction	0.274	73.91
*Ferula jaeschkeana* Vatke/Sib/SP-64	Apiaceae	Wild	Herb	Latex/Root powder	Asthma, arthritis and menstrual irregulation	Oral	Juice/decoction	0.357	80.00
*Carum carvi* L./Thalae/SP-63	Apiaceae	Wild	Herb	Seed	Asthma	Oral	Decoction	0.167	85.71
*Daucus carota* L./Wafro/SP-64	Apiaceae	Cult.	Herb	Root	Urethritis	Oral	Cook in water	0.214	77.77
*Artemisia brevifolia* Wall ex DC. /Bustay/SP-119	Asteraceae	Shrub	Herb	Leaves	Abdominal worms	Oral	Decoction	0.298	92.00
A*rtemisia santolinifolia* Turcz.ex Krasch./Kho bustae/SP-120	Asteraceae	Wild	Herb	Branches	Abdominal worms	Oral	decoction	0.167	71.42
*Tanacetum falconeri* Hook.f./Lhrtialo/SP-137	Asteraceae	Wild	Herb	Flower	Back ache, abdominal pain and gastric trouble	Oral	Decoction	0.476	95.00
*Taraxacum officinale* Weber /Shantha/SP-56	Asteraceae	Wild	Herb	Root	Hypertension	Oral	Boiled root	0.357	83.33
*Saussurea simpsoniana* (Fielding & Gardner) Lipsch./Smanipasha/SP-138	Asteraceae	Wild	Herb	Whole plant	Back ache, fever and dermatitis	Oral	Decoction	0.321	92.59
*Artemisia scoparia* Waldst. /Khasmar/SP-118	Asteraceae	Wild	Herb	Leaves	Diabetes	Oral	Infusion	0.143	75.00
*Berberis brandisiana* Ahrendt /Skiorbu/SP-150	Berberidaceae	Wild	Shrub	Leaves	Jaundice	Oral	Infusion	0.333	92.85
*Berberis orthrobotrys* Bien. ex Aitch. /Skiorbu/SP-94	Berberidaceae	Wild	Shrub	Leaves	Jaundice	Oral	Infusion	0.333	92.85
*Berberis pseudoumbellata* subsp. *gigitica* Jafri /Skiorbu/SP-60	Berberidaceae	Wild	Shrub	Seed	Jaundice	Oral	infusion	0.333	92.85
*Betula utilis* D.Don./Staqpa/SP-114	Betulaceae	Wild	Tree	Papery bark	Ringworm	Topical	Warm bark	0.190	93.75
*Biebersteinia odora* Steph. ex Fisch./Chundol/SP-69	Biebersteiniaceae	Wild	Herb	Flower	Migraine and fever	Oral	Decoction	0.202	94.11
*Onosma hispida* Wall. ex G.Don. /Kangmar/SP-50	Boraginaceae	Wild	Herb	Whole plant	Constipation	Oral	Fresh /Boiled is taken	0.226	89.47
*Myosotis alpestris* F.W.Schmidt /Mandaqskor/SP-47	Boraginaceae	Wild	Herb	Flower	Bronchitis, fever and asthma	Oral	Powder	0.357	90.00
*Arnebia guttata* Bunge /Thangmarsi/SP-138	Boraginaceae	Wild	Herb	Root	Heart burn and indigestion	Oral	Fresh root is taken	0.262	90.90
*Descurainia sophia* (L.) Webb & Berth./Khashir/SP-140	Brassicaceae	Wild	Herb	Seed	Fever	Oral	Decoction	0.167	57.14
*Brassica rapa* L./Mulo/SP-139	Brassicaceae	Wild	Herb	Root	Hepatitis	Oral	Fresh root is taken	0.298	60.00
*Capparis spinosa* Jafri/Traba/SP-147	Capparidaceae	Shrub	Shrub	Leaves	Arthritis and back ache	Oral	Decoction	0.214	83.33
*Kochia scoparia* (L.) Schard./Fiangma/SP-127	Chenopodiaceae	Wild	Herb	Leaves	Tiredness and hypertension	Oral	Decoction	0.167	35.71
*Juniperus excelsa* M.Bieb./Shukpa/SP-106	Cupressaceae	Wild	Tree	Fruit	Gastric trouble	Oral	Decoction	0.286	83.33
*Cuscuta epithymum* (L.) L./Rbulthaq/SP-79	Cuscutaceae	Wild	Herb	Stem	Asthma	Oral	Fresh material is taken	0.143	75.00
*Hippophe rhamnoides* subsp. *turkestanica* Rousiss/Karsoq/SP-70	Elaeagnaceae	Wild	Shrub	Fruit, Leaves	Hepatitis, hypertension and stomach ulcer	Oral	Fresh fruit is eaten	0.262	100.00
*Elaeagnus angustifolia* (L.) Kuntze/Sarsing/SP-91	Elaeagnaceae	Cult.	Tree	Fruit	Bronchitis	Oral	Decoction	0.238	85.00
*Ephedra gerardian*a Wall ex. Stapf. /Chae/SP-90	Ephedraceae	Wild	Shrub	Branches	Bronchitis, Vaginal contraction	Oral/Topical	Decoction	0.179	80.00
*Equisetum arvense* L. /Thangshiatwa/SP-77	Equisetaceae	Wild	Herb	Whole plant	Urethritis	Oral	Decoction	0.310	92.30
*Pisum sativum* L./Pokhstran/SP-86	Fabaceae	Cult.	Herb	Fruit	Constipation	Oral	Cooked fruits	0.238	55.00
*Trifolium repens* L./Skabuksuk/SP-81	Fabaceae	Wild	Herb	Leaves	Eye ache and wound	Topical	Fresh leaves	0.298	36.00
*Sophora alopecuroides* L. (Royle) Baker/Khakhul/SP-56	Fabaceae	Wild	Herb	Root	Joint pain	Oral	Decoction	0.214	83.33
*Desmodium gangeticum* L. /Shingnar/SP-73	Fabaceae	Wild	Shrub	Root	Cough, cold, asthma	Oral	Decoction	0.286	87.50
*Hedysarum falconeri* Baker /Kharun/SP-148	Fabaceae	Wild	Herb	Root	Loss of appetite	Oral	Fresh root is taken	0.190	87.50
*Vicia faba* L./Naqstarn/SP-82	Fabaceae	Cult.	Herb	Seed	Stomach ulcer	Oral	Seed is cooked	0.238	85.00
*Trigonella feonum-graecum* L./Shalmilik/SP-152	Fabaceae	Cult.	Herb	Leaves	Low blood pressure and gastric trouble	Oral	Fresh material with curd	0.226	78.94
*Corydalis adiantifolia* Hook.f. & Thomson/Shampoo/SP-49	Fumariaceae	Wild	Herb	Root	Hair tonic	Topical	Paste	0.286	87.50
*Swertia cordata* (G.Don) Clark/Tikta/SP-147	Gentianaceae	Wild	Herb	Whole plant	Diabetes	Oral	Decoction	0.262	90.90
*Ribes orientale* Desf./Askuta/Sp-57	Glossulariaceae	Wild	Shrub	Fruits	Abdominal worms	Oral	Eaten fresh	0.202	88.23
*Ribes himalense* Royle ex Decne./Askuta/SP-59	Glossulariaceae	Wild	Shrub	Fruit	Abdominal pain	Oral	Eaten fresh	0.250	95.23
*Ribes alpestre* Decne./Askuta/Sp-58	Glossulariaceae	Wild	Shrub	Fruit	Abdominal pain	Oral	Eaten fresh	0.262	68.18
*Juglans regia* L./Starga/SP-98	Juglandaceae	Cult.	Tree	Seed	Asthma	Oral	Fresh or dry seeds are eaten	0.179	66.66
*Dracocephalum nutans* L./Shundun/SP-76	Lamiaceae	Wild	Herb	Leaves	Asthma, gastric trouble post birth trace release	Oral	Decoction	0.262	86.36
*Coriandrum sativum* L. /Naqposhoto/SP-136	Lamiaceae	Cult.	Herb	Seed	Abdominal pain	Oral	Fresh or dry seeds are eaten	0.250	47.61
*Mentha royleana* Benth./Foling/SP-75	Lamiaceae	Wild	Herb	Leaves	Fever and gastric trouble	Oral	Decoction	0.262	81.81
*Nepeta leucolaena* Benth./Azumal/SP-149	Lamiaceae	Wild	Herb	Leaves	Indigestion and abdominal pain	Oral	Decoction	0.167	85.71
*Mentha arvensis* L./Piono/SP-78	Lamiaceae	Cult.	Herb	Leaves	Pimples and pustules	Oral	paste	0.357	83.33
*Thymus linearis* Benth./Tumburu/SP-45	Lamiaceae	Wild	Herb	Leaves	Cold and flue	Oral	Decoction	0.393	100.00
*Morus nigra* L./Osae/SP-113	Moraceae	Cult.	Tree	Fruit	Bronchitis, as blood tonic	Oral	Fresh material is eaten	0.238	85.00
*Fraxinus xanthoxyloides* (G.Don.) DC./Khara/SP-105	Oleaceae	Wild	Shrub	Stem/Seed	Ringworm and tooth ache	Oral	Fresh bark and seeds are applied	0.179	93.33
*Papaver nodicaule* L./Kialbumandoq/SP-126	Papaveraceae	Wild	Herb	flower	Wounds, cut and injuries	Topical	Powder	0.310	76.92
*Parnassia nubicola* Planch.ex Clark/Darbamandoq/SP-146	Parnassiaceae	Wild	Herb	Flower	Low blood pressure and gastric trouble	Oral	Decoction	0.286	33.33
*Plantago major* L./Bokhna/SP-115	Plantaginaceae	Wild	Herb	Flowers	Gastric trouble and constipation	Oral	Decoction	0.179	93.33
*Acantholimon lycopodioides* (Girad) Boiss./Choqmandoq/SP-68	Plumbaginaceae	Wild	Shrub	Flower	Stomach ulcer	Oral	Decoction	0.179	93.33
*Hordeum valgare* L./Nus/SP-154	Poaceae	Cult.	Herb	Seedlings	Stomach ulcer Hypertension	Oral	Decoction	0.167	85.71
*Triticum aestivum* L./Tro/SP-153	Poaceae	Wild	Herb	Seed	Constipation	Oral	Bread	0.262	81.81
*Zea mays* L./Makai/SP-155	Poaceae	Cult.	Herb	Carpels	Urethritis	Oral	Decoction	0.262	90.90
*Rumex nepalensis* Spreng./Bashona/145	Polygonaceae	Wild	Herb	Leaves	Pain and post birth trace release	Oral	Boiled material	0.190	68.75
*Fagopyrum esculentum* Moench, Meth. /Bro/SP-42	Polygonaceae	Cult.	Herb	Seed	Stomach ulcer	Oral	Powder	0.202	88.23
*Polygonum tataricum* L./Khobro/SP-144	Polygonaceae	Cult.	Herb	Seed	Stomach ulcer	Oral	Powder	0.190	87.50
*Rheum webbianum* Royle./Khol/Sp-121	Polygonaceae	Wild	Herb	Stem	Sole and lips crack	Oral/Topical	Fresh stem material/paste	0.226	84.21
*Rumex patientia* L./Rashona/SP-108	Polygonaceae	Wild	Herb	Leaves	Boils and pustules	Topical	Paste	0.238	85.00
*Punica granatum* L./Sio/SP-101	Punicaeae	Cult.	Tree	Fruit	Fever, vomiting	Oral	Eaten fresh	0.214	88.88
*Aconitum violaceum* var. *weileri* (Gilli) Riedl/Smunchan/SP-130	Ranunculaceae	Wild	Herb	Root	Abdominal worms	Oral	Paste	0.321	85.18
*Aconitum heterophyllum* Wall. ex Royle/Buma/SP-131	Ranunculaceae	Wild	Herb	Root	Abdominal worms and tooth ache	Oral/Topical	Decoction	0.179	66.66
*Clematis alpina* subsp. *sibirica* (L.) Kuntze/Ghnaghzima/SP-39	Ranunculaceae	Wild	Herb	Whole plant	Asthma	Oral	Decoction	0.167	85.71
*Ranunculus repens* L. /Khsiarmandoq/SP-132	Ranunculaceae	Wild	Herb	Flower	Ringworm	Topical	Paste	0.155	53.84
*Delphinium brunonianum* Royle /Makhoting/SP-88	Ranunculaceae	Wild	Herb	Flower	Hair tonic	Topical	Powder with oil	0.321	92.59
*Thalictrum foliolosum* DC. /Momiran/SP-133	Ranunculaceae	Wild	Herb	Root	Eye ache	Topical	Fresh material	0.262	54.54
*Prunus armeniaca* L./Chuli/SP-67	Rosaceae	Cult.	Tree	Fruit/Floral buds	Constipation and ringworm	Oral/Topical	Decoction and paste	0.155	69.23
*Rosa indica* L./Gulab/SP-134	Rosaceae	Cult.	Shrub	Flower	Fever and abdominal pain	Oral	Decoction	0.155	53.84
*Spiraeae canescens* D.Don./Khsiber/SP-54	Rosaceae	Wild	Shrub	Branches	Abdominal pain	Oral	Decoction	0.167	42.85
*Prunus avium* (L.) L/Gilas/SP-102	Rosaceae	Cult.	Tree	Fruit	Constipation	Oral	Cooked fruit	0.238	55.00
*Malus pumila* Mill./Kushu/SP-103	Rosaceae	Cult.	Tree	Fruit	Weakness, blood tonic	Oral	Eaten fresh	0.202	58.82
*Rosa webbiana* L./Sia/SP-71	Rosaceae	Wild	Shrub	Root Bark, Fruit	Hypertension, cold and flu	Oral	Decoction	0.286	95.83
*Salix alba* L./Hlchangma/SP-89	Salicaceae	Cult.	Tree	Leaves, Inflorescence	Post-birth bleeding, Lactation	Oral	Decoction	0.167	85.71
*Populus nigra* L./Naghbiar/SP-92	Salicaceae	Cult.	Tree	Bark	Jaundice, ring worm	Oral/Topical	Decoction	0.202	64.70
*Berginia ciliata* (Haw.) Sternb./Schapur/SP-43	Saxifragaceae	Wild	Herb	root	Stomach ulcer	Oral	Decoction	0.310	96.15
*Solanum nigrum* L./Drumbashokhlo/SP-156	Solanaceae	Wild	Herb	Fruit	Tooth ache	Oral	Fruits are toasted	0.179	86.66
*Datura fastuosa* L./Datura/SP-83	Solanaceae	Wild	Herb	Leaves	Pimples and pustules	Topical	Fresh material	0.155	76.92
*Urtica dioca* L./Khashoshing/SP-157	Urticaceae	Wild	Herb	Whole plant	Constipation	Oral	Cooked material	0.250	76.19
*Tribulus terrestris* L./Kokoring/SP-143	Zygophyllaceae	Wild	Herb	Fruit	Urethritis	Oral	Decoction	0.238	90.00

Present study illustrated diverse medicinal flora in the territories of Gilgit-Baltistan mountains. The exclusive alliance of medicinal plants, mountain restricted distribution and high level disagreement in traditional uses corroborate the significance of this study. Being the first inventory on medicinal flora of Shigar valley, present study offers baseline data for researchers, particularly interested in high mountains phyto-diversity and related traditional knowledge. The sub-alpine species in environs are practicable for conservation and cultivation [44, 50, 52]. The abundance of medicinal plant species in the study area could enhance the economic status of local communities by marketing and sustainable utilization. Local inhabitants can make their home gardens or micro park system of medicinally important species on their own land. However, illiteracy and lack of developmental packages are the major handicaps in the operation of such implications.

## Conclusion

The wealth of endemic and indigenous plant knowledge of the Balti community living in extremely isolated and high mountain Shigar valley, Karakorum ranges-Pakistan is reported for the first time. This study presented ethno-flora and traditional knowledge of the local inhabitants of the area. However, it would be in jeopardy; if further inclusive research is not conducted. Because mountain dwellers are oblivious of the values of bio-cultural diversity and the rate of transformation of plant knowledge decreasing with the passage of time due to infusing allopathic drugs and changing life style. Therefore, a comprehensive study in high mountain areas could be of significant value to conserve the medicinal plant wealth and related traditional knowledge. Moreover, extensive ethno-medicinal studies could discover the hidden knowledge and may provide unique plant species for chemical screening, consequently may leads to novel drugs discovery.
